# Effects of Cyanobacterial Lipopolysaccharides from *Microcystis* on Glutathione-Based Detoxification Pathways in the Zebrafish (*Danio rerio*) Embryo

**DOI:** 10.3390/toxins4060390

**Published:** 2012-05-25

**Authors:** Asha Jaja-Chimedza, Miroslav Gantar, Gregory D. Mayer, Patrick D. L. Gibbs, John P. Berry

**Affiliations:** 1 Department of Chemistry and Biochemistry, Marine Science Program, Florida International University, 3000 NE 151st Street, North Miami, FL 33181, USA; Email: ajaja001@fiu.edu; 2 Department of Biological Sciences, Florida International University, 11200 SW 8th Street, Miami, FL 33199, USA; Email: gantarm@fiu.edu; 3 The Institute for Environmental and Human Health, Texas Tech University, Lubbock, TX 79409, USA; Email: greg.mayer@ttu.edu; 4 Division of Marine Biology and Fisheries, Rosenstiel School of Marine and Atmospheric Science, University of Miami, 4600 Rickenbacker Causeway, Miami, FL 33149, USA; Email: pgibbs@rsmas.miami.edu

**Keywords:** lipopolysaccharide, cyanobacteria, zebrafish, *Microcystis*, glutathione, detoxification pathways, oxidative stress

## Abstract

Cyanobacteria (“blue-green algae”) are recognized producers of a diverse array of toxic secondary metabolites. Of these, the lipopolysaccharides (LPS), produced by all cyanobacteria, remain to be well investigated. In the current study, we specifically employed the zebrafish (*Danio rerio*) embryo to investigate the effects of LPS from geographically diverse strains of the widespread cyanobacterial genus, *Microcystis*, on several detoxifying enzymes/pathways, including glutathione-S-transferase (GST), glutathione peroxidase (GPx)/glutathione reductase (GR), superoxide dismutase (SOD), and catalase (CAT), and compared observed effects to those of heterotrophic bacterial (*i.e.*, *E. coli*) LPS. In agreement with previous studies, cyanobacterial LPS significantly reduced GST in embryos exposed to LPS in all treatments. In contrast, GPx moderately increased in embryos exposed to LPS, with no effect on reciprocal GR activity. Interestingly, total glutathione levels were elevated in embryos exposed to *Microcystis* LPS, but the relative levels of reduced and oxidized glutathione (*i.e.*, GSH/GSSG) were, likewise, elevated suggesting that oxidative stress is not involved in the observed effects as typical of heterotrophic bacterial LPS in mammalian systems. In further support of this, no effect was observed with respect to CAT or SOD activity. These findings demonstrate that *Microcystis* LPS affects glutathione-based detoxification pathways in the zebrafish embryo, and more generally, that this model is well suited for investigating the apparent toxicophore of cyanobacterial LPS, including possible differences in structure-activity relationships between heterotrophic and cyanobacterial LPS, and teleost fish *versus* mammalian systems.

## 1. Introduction

Cyanobacteria, also known as “blue-green algae”, are photosynthetic bacteria that, although being ubiquitous in their distribution, are perhaps best studied in freshwater systems where they can form large, noxious and potentially toxigenic blooms. Cyanobacteria are well known to produce a diversity of toxic secondary metabolites, including hepatotoxins (e.g., microcystins, cylindrospermopsin), neurotoxins (e.g., saxitoxin, anatoxin-a), cytotoxins, dermatotoxins (e.g., lyngbyatoxin) and “endotoxins” (*i.e.*, lipopolysaccharides), and have consequently become a growing environmental health concern (see, for example, [1]). Of the known cyanobacterial toxins, the chemically complex lipopolysaccharides (LPS) remain among the least understood, despite the fact that they are produced by all cyanobacteria.

LPS, in general, are characteristic structural components of the outer membrane of Gram-negative bacteria (including cyanobacteria), and, in the case of heterotrophic representatives, are well described as the so-called “endotoxins” associated with sepsis and related toxicoses associated with bacterial infection. Indeed, the vast majority of what is known about LPS chemistry and toxicity has been exclusively described for heterotrophic bacterial endotoxin. Structurally, LPS described from heterotrophic bacteria [2] are comprised of a lipid moiety, lipid A, typically characterized by acylated and phosphorylated glucosamine disaccharides, attached to a polysaccharide backbone, including an oligosaccharide “core”, generally characterized by heptose and 3-deoxy-D-mannooctulosonic acid (Kdo), and a variable outer polysaccharide (“O-antigen”). Toxicologically, heterotrophic bacterial LPS are primarily recognized as pro-inflammatory and immunomodulatory molecules, eliciting expression of various signaling molecules (e.g., cytokines/chemokines, eicosanoids), and release of superoxide radicals and other reactive oxygen species (ROSs), leading to oxidative stress [3,4]. Specifically, it has been well established that LPS initiation of these activities is centrally mediated via interactions between lipid A and toll-like receptor 4 (TLR4), a member of the TLR family of transmembrane proteins [5,6].

Recent research has suggested that cyanobacterial LPS may be linked to a wide variety of health effects in humans, including gastro-intestinal illness, skin rashes, allergy, respiratory disease, headache, fever, and other pathologies, however, limited studies have investigated possible mechanisms associated with these effects [7]. In recent studies [8], LPS from the cyanobacterial species, *Microcystis aeruginosa*, was shown to stimulate the production/release of neuroinflammation mediators, including superoxide radicals, cytokines/chemokines and eicosanoids, and various related proteins in rat brain microglia, but activities were generally only observed at considerably higher (*i.e.*, 10^2^–10^3^ fold) concentrations when compared to heterotrophic bacterial LPS. 

In the present study, the zebrafish (*Danio rerio*) embryo model was used to investigate the effects of the cyanobacterial LPS on detoxifying enzymes. For the past decade or more, the zebrafish model has been increasingly used in a growing number of research areas such as toxicological studies [9,10,11], genetic studies of development and human disease [12,13], and drug screening and discovery [14]. Among various practical advantages, zebrafish are relatively easy to maintain and breed, and the embryos are transparent and fully develop most organ systems within 96 h post-fertilization (hpf), making them an ideal vertebrate model system. With respect to the current study, the zebrafish embryo has been used previously to investigate cyanobacterial toxins generally [10,11], and cyanobacterial LPS specifically [15,16].

It has been generally established [17,18] that lower invertebrates, including fish, are (compared to mammalian systems) relatively resistant to heterotrophic bacterial LPS by conventional measures (e.g., activation of immune cells). Specifically, it has been recently suggested [19] that resistance of fish to heterotrophic bacterial LPS is likely due to evolutionary divergence of the TLR4 receptor (*i.e.*, the “LPS receptor” in mammals). In contrast, however, a prior study by Best *et al.*, [15] investigated the effects of cyanobacterial LPS on the detoxifying GST enyzme in embryonic *D. rerio*, and found that LPS from various cyanobacterial sources reduced the activity of both soluble and microsomal GST in *D. rerio* embryos within 24 hpf. Moreover, inhibition of GST by cyanobacterial LPS was significantly higher compared to heterotrophic bacterial (e.g., *Escherichia coli*, *Salmonella typhimurium*) LPS. These findings, therefore, suggest not only that yet uncharacterized chemical features of cyanobacterial LPS may present unique (from other bacterial) toxicophores, but also that teleost fish (such as zebrafish) represent a unique model for evaluating these compounds.

The current study focused on the effect of LPS from several, geographically diverse strains of the widespread genus, *Microcystis*, on several detoxification enzymes/pathways in the zebrafish embryo. Specifically we evaluated glutathione-based pathways, GST and glutathione peroxidase (GPx)/glutathione reductase (GR), as well as superoxide dismutase (SOD) and catalase (CAT) that interact directly with ROSs. To further assess the role of oxidative stress, we evaluated both the total glutathione pool, as well as exclusive and relative concentrations of reduced/oxidative glutathione (GSH/GSSG) levels. 

## 2. Materials and Methods

### 2.1. *Microcystis* Culture and LPS Extraction

Strains of cyanobacteria belonging to the genus, *Microcystis*, were cultured in the Department of Biological Sciences at Florida International University (Miami, FL). Strains included those isolated from temperate freshwater systems of the Great Lakes, MC 81-11 (Bay of Quinte, Lake Ontario) and MC 95-11 (Lake Erie), and from sub-tropical South Florida, MC 36-1 (Doctors Lake, Hog Point, FL, USA), as well as a strain (299) of *M. aeruginosa* obtained from the reference collection of the University of Toronto Culture Collection of algae and cyanobacteria (Table 1). All isolates (except *M. aeruginosa* 299) were taxonomically identified (to genus) by microscopic observations using classical morphological criteria given in Komarek and Anagnostidis [20,21]. The cultures were grown in 3L flasks in BG11 medium under cool-white fluorescent light (intensity 30 µEm^−2^s^−1^) with aeration as previously described [10,11,22]. The cells were harvested after three to four weeks by centrifugation and freeze-dried. 

LPS were extracted from collected cyanobacterial biomass using the standard “hot phenol-water extraction” method as modified by Bernadova *et al.* [23]. Briefly, freeze-dried biomass from cultures were re-suspended in pyrogen-free water, added to equal volume of 90% phenol (in water) and extracted (with stirring) at 68 °C for 20 min. The aqueous layer was removed by centrifugation (2500 *g* for 30 min), and the remaining phenol extracted a second time with water. The pooled aqueous fractions were dialyzed (3500 molecular weight cut-off cellulose) against distilled water, and lyophilized. The lyophilized sample was re-dissolved in 0.1 M Tris buffer with 25 µg·mL^−1^ RnaseA (Sigma-Aldrich, St. Louis, MO, USA), and incubated at 37 °C for 16 h. An equal volume of phenol was added to the resuspended sample, and after incubation (at room temperature for 4 min), the aqueous layer was collected by centrifugation, dialyzed a second time and subsequently lyophilized to produce the LPS in powder form. In addition to the LPS extracted from cyanobacterial biomass, a standard of LPS from *Escherichia coli* (0128:B12), a heterotrophic bacterium, was purchased from Sigma-Aldrich (St. Louis, MO, USA).

### 2.2. Endotoxin Activity and Microcystin Content of LPS Extractions

“Endotoxin activity” is a commonly employed measure of LPS, particularly in the investigation of heterotrophic bacteria, and was used here to identify possible correlation between this activity and other biological activities. Specifically, we utilized the standard Limulus Amoebocyte Lysate (LAL) Endosafe Endochrome-K Assay that gives a quantitative detection of endotoxins by kinetic-chromogenic methods. Analyses were performed by Charles River Laboratories, Inc. (Wilmington, MA). Endotoxin activity was expressed as endotoxin unit per mg of LPS (EU/mg). In addition to endotoxin analysis, an enzyme-linked immunosorbent assay (ELISA) specific to the widespread microcystins (MCYSTs), and related nodularins (Abraxis Kits, Warminster, PA), was performed to test for MCYST content in the prepared LPS, and eliminate any possible contribution of this cyanobacterial toxin.

### 2.3. Breeding and Maintenance of Zebrafish

Zebrafish embryos of the L3 line (obtained from the University of Miami Rosenstiel School of Marine and Atmospheric Science-RSMAS) were used for all experiments presented here. Maintenance and breeding of the zebrafish were carried out by standard methods, and specifically as described by Berry *et al*. [10,11]. After successful breeding, the eggs were collected, rinsed and placed in a Petri dish with E3 medium (5 mM NaCl, 0.17 mM KCl, 0.33 mM CaCl_2_, and 0.33 mM MgSO_4_; [24]). Unfertilized and poor quality (*i.e.*, clearly moribund) embryos were removed from the dish and the remaining embryos were subsequently used within 3 hpf (4- to 64-cell stage) for exposures.

### 2.4. Exposure Studies and Enzyme Assays

For evaluation of detoxifying enzymes, embryos were exposed to 250 μg/L of LPS from *E. coli* and *Microcystis* isolates in E3 medium based on a method modified from Cazenave *et al.* [25] for 24 h. Specifically, 200 embryos per replicate were placed in polystyrene culture dishes, containing LPS (diluted to 250 µg/L) in 30 mL E3 medium; for negative controls, embryos were placed in dishes containing 30 mL of E3 medium only. All exposures were done in triplicate (600 embryos total). The embryos were then “flash frozen” in liquid nitrogen and stored at −80 °C until enzyme extraction. The enzyme preparations were carried out according to the methods of Wiegand *et al*. [26] by which the samples were mechanically homogenized using phosphate buffer at a pH of 7.4. The cell debris was removed, and the supernatant was collected for enzyme studies. To normalize enzyme activity, total protein content was determined by the “Bradford Assay” [27] using bovine serum albumin as a standard.

Enzyme (GST, GPx, GR, SOD and CAT) activities, as well as total glutathione and relative reduced/oxidized glutathione (GSH/GSSG) were evaluated using commercially available kits (Sigma-Aldrich, St. Louis, MO; Cayman Chemical Company, Ann Arbor, MI), as per manufacturer’s instructions and standard techniques, and expressed relative to total protein. Spectrophotometric measurements were made using a Biotek Synergy HT multi-well plate reader. Total glutathione was determined based on spectrophotometric measure (412 nm) of the production of the 5-thio-2-nitrobenzoic acid (TNB) product; samples were deproteinated using metaphosphoric acid and triethanolamine prior to assays. Oxidized glutathione (GSSG) was specifically determined by derivatizing reduced GSH using 2-vinylpyridine (prior to assays, but following deproteination). GST activity (µmol/mL/min) was measured colorimetrically, specifically based on the conjugation of GSH to 1-chloro-2, 4-dinitrobenzene (CDNB), and subsequent measurement of the product at 340 nm. GPx was measured based on the decrease in NAPDH (340 nm) that is consumed in the recycling of oxidized GSSG to GSH, following reduction of hydroperoxide. GR, on the other hand, was determined, based on the conjugation of GSH to 5,5′-dithiobis(2-nitrobenzoic acid) following recycling of GSSG to the reduced form by GR. Both GPx and GR activity of embryo preparations were compared to standards of purified enzymes. CAT and SOD activity were both determined colorimetrically using kits from Cayman Chemical Company; CAT assays specifically measured the production of formaldehyde (from methanol) using the chromagen, 4-amino-3-hydrazino-5-mercapto-1,2,4-triazole (540 nm), whereas SOD activity was measured based on the dismutation of superoxide radicals, generated by xanthine oxidase, using tetrazolium salts (440 nm).

### 2.5. Statistics

For the enzymes activities assays, a one-way ANOVA followed by F-test was carried out to determine if LPS treatments were statistically different from the control (*p* ≤ 0.05). 

## 3. Results and Discussion

### 3.1. LPS from *Microcystis*

Cyanobacterial LPS were isolated from laboratory cultures of geographically diverse *Microcystis* strains, including temperate (MC 81-11 and MC 95-11) and sub-tropical (MC 36-1) representatives. LPS were found to account for up to 4.1% of the dried biomass weight (Table 1). In general agreement with prior studies of heterotrophic bacterial [28] and cyanobacterial [29] LPS, considerable variation in extraction yield (0.1–4.1%) between *Microcystis* strains was observed (Table 1). In fact, LPS from an additional sub-tropical *Microcystis* isolate were prepared, but yielded too little for subsequent experimental studies (data not shown).

**Table 1:** Extraction yield of LPS prepared from *Microcystis* isolates, and the endotoxin activity (in endotoxin units [EU/mg]) of these preparations, used in the current study.

Cyanobacterial Strain	Source	% Yield of LPS	Endotoxin Activity (×10^3^ EU/mg)
*M. aeruginosa* 299	UTCC ^a^	0.1	107.5
MC 36-1	Doctors Lake, FL	0.4	0.1619
MC 81-11	Lake Ontario	4.1	36.65
MC 95-11	Lake Erie	2.4	6.327

^a^ University of Toronto Culture Collection of algae and cyanobacteria.

Endotoxin activities of LPS preparations were measured, using the standard LAL assay, and found to be highly variable (Table 1). Specifically, endotoxin activity (per unit weight, *i.e.*, EU/mg) associated with LPS preparations varied over nearly three orders of magnitude. This variability in endotoxin activity agrees with previous studies [23,30,31] in which the LPS from several, diverse cyanobacterial species were studied, and the endotoxin activities, based on the *Limulus* assay, likewise, were seen to vary considerably (*i.e.*, 2–3 orders of magnitude). 

Indeed, compared to heterotrophic bacterial LPS, little is known about the structural and chemical composition of cyanobacterial LPS, in general, and specifically how chemical composition/structure differences may relate to endotoxin activity. Raziuddin *et al*. [32] compared LPS from two strains of *Microcystis aeruginosa* to *Salmonella arbortus equi*, and found that cyanobacterial LPS had considerably less endotoxin activity than the heterotrophic bacteria (based the LAL assay). More generally, cyanobacterial LPS have been found to be less active in all of the models typically associated with endotoxin activity [8,30]. 

Only one recent study [33] has provided a complete structural elucidation of a cyanobacterial LPS. Specifically, Snyder *et al*. [33] characterized the structure of LPS from a marine cyanobacterial genus, *Synechococcus*. In this study, cyanobacterial LPS (compared to typical heterotrophic bacterial LPS) was found to represent a simplified, and possibly “primordial,” structure. In particular, *Synechococcus* LPS lacked the usual heptose and Kdo-replaced, instead, by 4-linked glucose-in the oligosaccharide core, and contained a lipid A moiety uniquely characterized by odd-chain hydroxylated fatty acids and lack of phosphates. Similarly, Lindsay *et al*. [29] recently evaluated LPS preparations from several laboratory strains of cyanobacteria, including *Microcystis*, and did not detect Kdo in any of the samples evaluated. Unlike preparations from *Microcystis*, LPS from *Synechococcus* showed no LAL activity. Taken together with existing toxicological information, these findings strongly suggest that chemical differences relative to heterotrophic bacterial LPS- and particularly, perhaps, the lipid A structure (associated with TLR4-mediated effects; see *Introduction*) or oligosaccharide core-may underlie the relatively low endotoxin-like activity of cyanobacterial LPS.

In addition to LPS, *Microcystis* is a well-known producer of the heptapeptide toxin, microcystin. Microcystins have been linked to modulation of each of the detoxification pathways investigated in the present study [34,35,36,37]. Evaluation of LPS preparation using ELISA, specific to the characteristic Adda moiety of microcystins (and related nodularins), suggested that these toxins were not present (within the limit of quantitation, *i.e.*, ≥0.15 ppb) in any of the samples evaluated, and thus not associated with observed effects on detoxification pathways (discussed below). 

### 3.2. Effects of *Microcystis* LPS on Detoxification Pathways in the Zebrafish Embryo

Relative to heterotrophic bacterial LPS, there have been very limited studies on the toxicology of cyanobacterial LPS. In one very recent study, specifically using LPS isolated from one of the same cyanobacterial strains (*i.e.*, *M.aeruginosa* 299) investigated here, Mayer *et al*. [8] showed that cyanobacterial LPS modulates expression of several proinflammatory markers in the rat brain microglia model. However, effects were typically observed at two to three orders of magnitude higher concentrations for cyanobacterial, compared to heterotrophic bacterial, LPS. Similarly, Keleti and Sykora [30] previously showed that cyanobacterial LPS from *Oscillatoria brevis* and *Anabaena cylindrical* are about ten times less toxic, in adrenalectomized mice, than heterotrophic bacterial (*i.e.*, *Salmonella*) LPS.

In addition to these studies, several recent studies (including the present) have specifically investigated the effects of cyanobacterial LPS in various teleost fish models [15,16,37,38]. Unlike mammalian models, teleost fish have been found to either lack, or contain an evolutionarily divergent form of, TLR4 [19] as the recognized mediator of endotoxin, e.g., immunomodulatory, activity [39]. Moreover, this evolutionary divergence of TLR4 has been clearly linked to the resistance of teleost fish to the immunomodulatory effects of heterotrophic bacterial LPS [17,18]. For example, Sepulcre *et al*. [18] showed that, although heterotrophic bacterial LPS stimulated immunomodulatory effects in zebrafish (which express an evolutionarily divergent TLR4 ortholog, whereas more evolutionarily advanced fish do not), these effects were only observed at significantly (approximately 10^3^-fold) higher doses of LPS, and occurred via TLR4-independent pathways.

In the present study, we evaluated the effects of LPS from *Microcystis* in relation to several detoxification enzymes/pathways in the zebrafish embryo model. No difference in mortality, or apparent impairment of development, was observed in zebrafish embryos exposed to any of the LPS treatments. However, a significant modulation of several detoxifying pathways was measured. 

**Figure 1:**
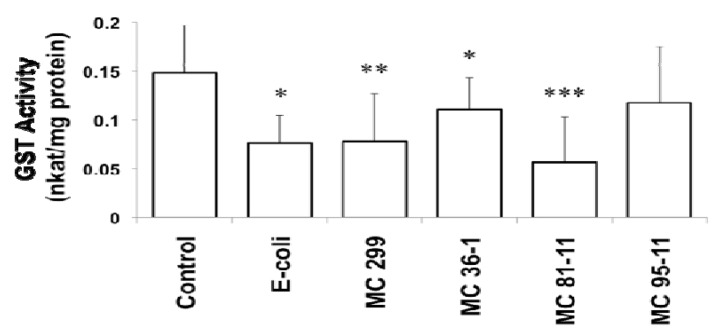
Effect of LPS on Glutathione*-*S-Transferase (GST) activity in 24 hpf zebrafish embryos. Embryos were exposed to cyanobacterial and heterotrophic bacterial LPS at concentrations of 250 µg/L for 24 h. An asterisk signifies the GST activity that is significantly different from the control (* for *p* < 0.05, ** for *p* < 0.01, and *** for *p* < 0.001). Columns represent the mean of eight measurements. Error bars represent the standard deviation from the mean.

GST activity decreased after 24 h exposure of zebrafish embryos to both heterotrophic bacterial and cyanobacterial LPS (at 250 µg/L). Compared to the control group (0.15 nkatal/mg protein), GST activity was significantly decreased in embryos that were exposed to *E. coli* (0.076 nkatal/mg protein), *M. aeruginosa* 299 (0.078 nkatal/mg protein), MC 36-1 (0.11 nkatal/mg protein) and MC 81-11 (0.056 nkatal/mg protein) LPS. Embryos exposed to MC 95-11 LPS showed a decrease (0.12 nkatal/mg protein), although it was not statistically significant (Figure 1). Inhibition of GST (compared to “No LPS” controls) varied considerably between LPS preparations, ranging 61.9% inhibition for the most active (MC 81-11) to approximately 20–25% for the least active (20.6% and 25.5%, respectively for MC 95-11 and MC 36-1). 

Inhibition of GST is consistent with prior studies in zebrafish and other teleost fish models [15,37,38]. Best *et al*. [15] similarly demonstrated GST inhibition by cyaonbacterial LPS in the zebrafish embryo model. More recently, Wang *et al*. [37] found apparent down-regulation of GST mRNA in response to LPS in the Nile tilapia (*Oreochromis niloticus*). 

**Figure 2:**
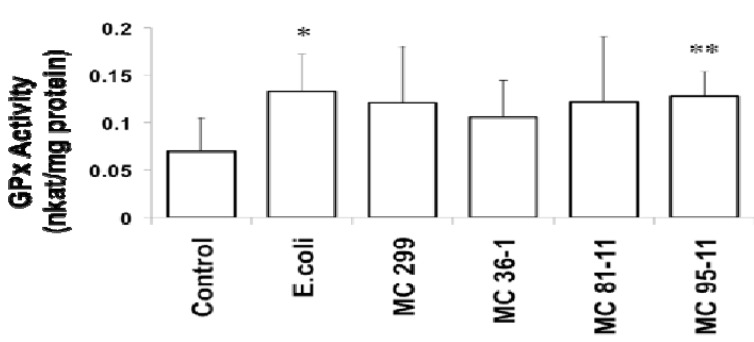
Effect of LPS on Glutathione Peroxidase (GPx) in 24 hpf zebrafish embryos. Embryos were exposed to cyanobacterial and heterotrophic bacterial LPS at concentrations of 250 µg/L for 24 h. An asterisk denotes significant difference from the control (* for *p* < 0.05 and ** for *p* < 0.01). Columns represent the mean of six measurements. Error bars represent the standard deviation from the mean.

In addition to GST, we also investigated two additional enzymes, GPx and GR, which reciprocally utilize glutathione, and specifically recycle the peptide between its reduced and oxidized forms. While no effects of LPS on GR were observed, GPx moderately increased in zebrafish embryos exposed to all LPS evaluated (Figure 2). GPx showed a significant increase from the controls (0.07 nkatal/mg protein) in embryos exposed to *E. coli* (0.13 nkatal/mg protein), and MC 95-11 (0.13 nkatal/mg protein). Though increases in the embryos exposed to *M. aeruginosa* 299 (0.12 nkatal/mg protein), MC 36-1 (0.11 nkatal/mg protein), and MC 81-11 (0.12 nkatal/mg protein) were observed, they were not statistically significant (Figure 2). To the authors’ knowledge, this is the first report of the effect of LPS on GPx in the zebrafish embryo model. However, this is consistent with prior studies of tilapia by Wang *et al*. [37] who, likewise, showed a moderate up-regulation of GPx transcription by LPS. 

**Figure 3:**
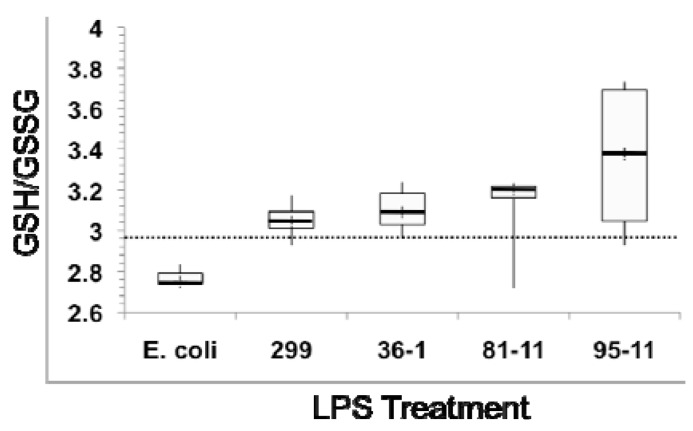
Effects of LPS exposure on ratio of reduced (GSH) to oxidized (GSSG) glutathione as a measure of oxidative stress in 24 hpf zebrafish embryos. Dotted line represents GSH/GSSG of untreated (“No LPS”) controls. Box plots show minimum (lower whisker) and maximum (upper whisker), as well as 1st and 3rd quartile (bottom and top of box, respectively), and median (central thicker line), of the calculated ratio for six measurements.

In order to evaluate the role of oxidative stress, as well as the possible effects of LPS on glutathione levels, in relation to the modulation of GST and GPx, we measured both total glutathione, as well as relative ratios of reduced (GSH) and oxidized (GSSG) forms. Heterotrophic bacterial LPS is associated with oxidative stress in multiple model systems [40]. The relative concentration of GSH/GSSG, and specifically a decrease in this ratio—due to the relative depletion of GSH, and subsequent accumulation of the oxidized GSSG—has been widely employed as an indicator of oxidative stress in a range of organisms [41], including zebrafish [42]. In agreement with this, we observed a slight, but not statistically significant, decrease in GSH/GSSG ratio (Figure 3) in embryos exposed to *E. coli* LPS (Figure 3). The slightness of the effect would be consistent with prior studies [19] which indicate teleost fish, and specifically zebrafish, are generally resistant to TLR4-mediated effects (e.g., immunomodulatory, oxidative stress) of heterotrophic bacteria LPS. 

In contrast, we actually observed an increase, although not statistically significant, in the ratio of reduced/oxidized glutathione in embryos exposed to cyanobacterial LPS (Figure 3), suggesting an alternative mechanism, and not oxidiative stress, for concomitant modulation of GST and GPx (see below). In further support of this, no change in either SOD or CAT activity was observed (data not shown) in embryos exposed to LPS. Unlike GST and GPx that primarily act via interaction with ROS-generating xenobiotics or products of oxidative damage (e.g., lipid peroxides), both SOD and CAT directly detoxify ROSs (*i.e.*, superoxide and hydrogen peroxide, respectively), and are thus generally associated most directly with oxidative stress. 

**Figure 4:**
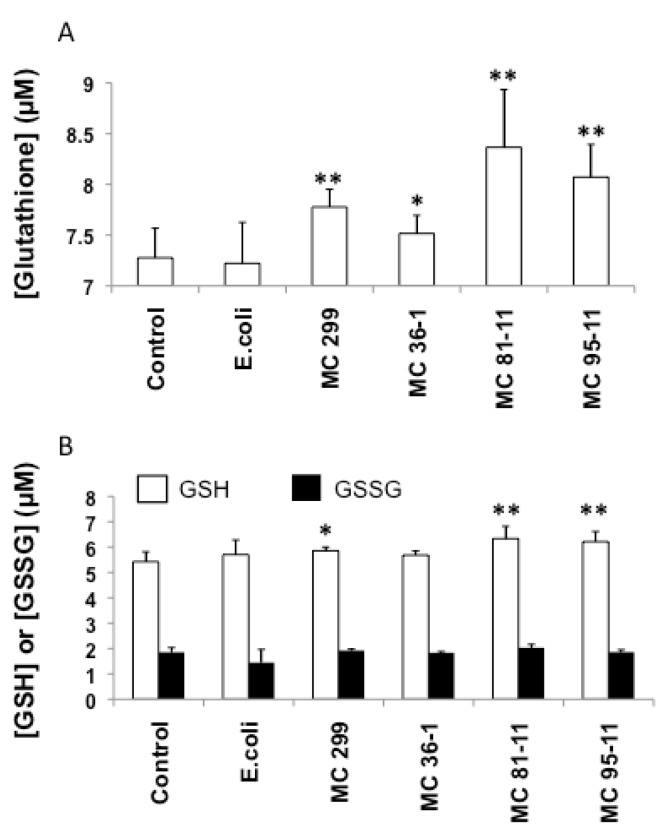
Effects of LPS on total glutathione (**A**), and exclusive measurements of reduced (GSH, open columns) and oxidized (GSSG, black columns) glutathione (**B**) in 24 hpf zebrafish embryos. Embryos were exposed to cyanobacterial and heterotrophic bacterial LPS over a 24 h period. Columns represent the mean of six measurements. Error bars represent the standard deviation from the mean. An asterisk denotes a significant difference from the control (* denotes *p* < 0.05, and ** denotes *p* < 0.01).

Rather than oxidative stress, cyanobacterial LPS increased both total glutathione and reduced GSH (Figure 4) levels in exposed embryos. Embryos exposed to all cyanobacterial LPS treatments showed a significant increase total glutathione pool (*i.e.*, both GSH and GSSG; Figure 4A), relative to the untreated (*i.e.*, “No LPS”) controls (7.28 µM). In contrast, there was no significant increase-and, in fact, a slight decrease (7.22 µM)-in total glutathione for embryos exposed to *E. coli* LPS (Figure 4A). Exclusive measurements of reduced and oxidized glutathione determined that GSSG content showed no significant difference compared to the control, but a significant increase in GSH content for embryos exposed to LPS from *M. aeruginosa* 299 (5.86 µM, *p* < 0.05), MC 81-11 (6.33 µM, *p* < 0.01), and MC 95-11 (6.21 µM, *p* < 0.01) (Figure 4B). GSH levels also increased, relative to controls (5.42 µM) in embryos exposed to LPS from MC 36-1 (5.68 µM), as well as *E. coli* (5.70 µM), but increases were not statistically significant. 

These results, taken together, support an alternative mechanism (*i.e.*, not oxidative stress), and perhaps a possible direct interaction between LPS and glutathione-based detoxification pathways. The increase in total glutathione pool (Figure 4A) clearly suggests that cyanobacterial LPS either directly or indirectly elevate levels of the peptide. In support of a direct interaction between LPS and GST expression, and consequent activity, Wang *et al*. [37] cloned the putative soluble GST-α gene from tilapia—the transcription of which was shown to increase with LPS exposure-and identified a putative “LPS response element.” Comparison to the sequence of a GST-α (BC056725) cloned from zebrafish, in fact, identifies a similar sequence, and further supports the possible involvement of transcription level regulation of GST by LPS in this model. Likewise, the same study showed a concomitant increase in GPx transcription (in the trout model) with LPS exposure, in agreement with moderate effects on GPx activity observed here.

## 4. Conclusions

Although cyanobacterial LPS are ubiquitous, particularly in freshwater habitats, very few toxicological studies have investigated their relevant bioactivities (particularly relative to heterotrophic bacterial “endotoxin”). In the current study, we utilized the zebrafish embryo, as a toxicological model, to investigate the effects of LPS from the widespread cyanobacterial genus, *Microcystis*, on several detoxification enzymes/pathways.

These studies demonstrate that cyanobacterial LPS modulate glutathione-based detoxification pathways including apparent inhibition of GST activity, stimulation of GPx activity and elevation of total and reduced glutathione. Moreover, whereas a possible role of oxidative stress (as evidenced by reduced GSH/GSSG ratio) was suggested for heterotrophic bacterial LPS, an observed increase in GSH and GSH/GSSG ratio in zebrafish embryos exposed to cyanobacterial LPS suggests an alternative mechanism, and perhaps a direct interaction with glutathione-based enzymes/pathways. In the case of GST and GPx, this is supported by prior studies in tilapia [37] that documented transcriptional level effects of LPS on both enzymes. On the other hand, possible mechanisms for the observed increase in glutathione levels, including direct effects (e.g., stimulation of glutathione biosynthesis) or indirect effects (e.g., accumulation of GSH associated with GST inhibition), remain to be investigated. Moreover, the alternative mechanism suggested here (*i.e.*, interaction with glutathione-based pathways), specifically in relation to previously identified differences between heterotrophic and cyanobacterial LPS chemical composition (e.g., lipid A and lack of Kdo; [29,33]), and between mammalian and teleost fish systems (*i.e.*, absence of TLR4 in the latter; [19]), more generally indicates a possibly novel toxicophore for cyanbacterial LPS in teleost fish. Further chemical characterization of cyanobacterial LPS, in relation to relevant activities, are needed to address this possibility.

Finally, the observed effects of LPS on glutathione-based detoxification pathways may explain previously observed interactive effects of LPS with other toxicants. In particular, cyanobacteria produce a wide array of toxic or otherwise bioactive metabolites, including several recognized toxins (e.g., microcystins, cylindrospermopsin, anatoxin-a, saxitoxin). In the case of the widespread hepatotoxin, microcystin (also produced by *Microcystis*), for example, detoxification has been clearly linked to GST conjugation of GSH to the toxin [43,44,45]. As such, modulation of these detoxification pathways by LPS could present an interactive role with toxicity. In agreement with this, Best *et al.* [46] more recently found that co-exposure of trout to microcystin and LPS significantly increased the toxicity of the former (as measured by hepatosomatic index). It is proposed that such a finding could be explained by, for example, reduced detoxification capacity (e.g., GST inhibition) associated with LPS exposure. Similarly, interactive effects between LPS and cyanobacterial toxins, including microcystins and cylindrospermopsin, have been observed in aquatic invertebrates, and particularly *Daphnia* [47,48]. In the case of cylindrospermopsin, it has been shown that the toxin actually inhibits synthesis of reduced GSH [49], and as mentioned previously, microcystin detoxification is recognized to involve GST conjugation [43,44,45]. Interestingly, however, it was shown that co-exposure, in the case of *Daphnia*, to cyanobacterial LPS and microcystin or cylindrospermopsin had, in fact, a protective effect against these latter toxins. An interactive role would be obviously relevant to toxicological studies of these cyanobacterial toxins as, in natural settings (e.g., freshwater systems), organisms would be exposed to not only the toxins, but cellular LPS (as components of all cyanobacterial cell walls). However, further studies are required to clarify the complexities of these possible interactions.
